# Comparative effects of insulin-like growth factor-1, lycopene, and α-tocopherol on mitochondrial dynamics and developmental competence of buffalo (*Bubalus bubalis*) oocytes during *in vitro* maturation

**DOI:** 10.14202/vetworld.2026.1178-1195

**Published:** 2026-03-23

**Authors:** Omaima Mohamed Kandil, Sara Mohamed Elamey, Sayed Ahmed Hattab, Nabil Mohamed Baker, Mohamed Asran Elbehiry

**Affiliations:** 1 Department of Animal Reproduction and Artificial Insemination, Veterinary Research Institute, Center of Excellence, Embryo and Genetic Resources Conservation Bank, National Research Centre, Cairo, Egypt; 2 Department of Theriogenology, Faculty of Veterinary Medicine, Damanhour University, Elgomhoreia street, Damanhour, Egypt; 3 Department of Theriogenology, Faculty of Veterinary Medicine, Alexandria University, Moharram Bek, Alexandria, Egypt; 4 Department of Infectious Diseases, Faculty of Veterinary Medicine, Damanhour University, Elgomhoreia street, Damanhour, Egypt

**Keywords:** buffalo oocytes, embryo development, insulin-like growth factor-1, *in vitro* maturation, lycopene, mitochondrial dynamics, mitochondrial function, α-tocopherol

## Abstract

**Background and Aim:**

In *in vitro* embryo production (IVEP) systems, the efficiency of oocyte maturation and subsequent embryo development is often limited by oxidative stress and suboptimal mitochondrial function. Supplementation of maturation media with growth factors and antioxidants has been proposed as a strategy to enhance oocyte developmental competence. Insulin-like growth factor-1 (IGF-1) promotes cell survival and proliferation, while antioxidants such as lycopene and α-tocopherol reduce intracellular reactive oxygen species (ROS) and protect cellular structures from oxidative damage. Although these supplements have individually demonstrated beneficial effects in various species, comparative studies evaluating their influence under identical conditions in buffalo (*Bubalus bubalis*) oocytes are limited. Therefore, this study aimed to evaluate and compare the effects of IGF-1, lycopene, and α-tocopherol supplementation during *in vitro* maturation on nuclear maturation, embryo developmental competence, and mitochondrial dynamics in buffalo oocytes.

**Materials and Methods:**

A total of 1,485 high-quality buffalo oocytes were subjected to *in vitro* maturation (IVM) in four experimental groups: control (Tissue culture medium [TCM]-199), TCM-199 supplemented with 100 ng/mL IGF-1, TCM-199 supplemented with 0.2 μM lycopene, and TCM-199 supplemented with 100 μM α-tocopherol. Oocytes were incubated for 22 h at 38.5°C under 5% CO_2_. Mature oocytes (n = 1,149) were then fertilized *in vitro* using Fert-TALP medium and cultured in modified synthetic oviductal fluid (mSOF) for 7 days to evaluate cleavage, morula, and blastocyst formation rates. Mitochondrial activity and distribution were assessed in 120 mature oocytes using MitoTracker Red FM staining followed by confocal laser scanning microscopy. Mitochondrial patterns were classified as diffuse, semi-diffuse, semi-peripheral, or peripheral. Data were analyzed using one-way analysis of variance followed by Tukey’s post hoc test or chi-square analysis, with significance set at p ≤ 0.05.

**Results:**

The nuclear maturation rate (metaphase II stage) was significantly higher (p < 0.01) in the IGF-1 and lycopene groups (85.2% and 87.3%, respectively) compared with the control (73.3%) and α-tocopherol groups (76.2%). Cleavage, morula, and blastocyst formation rates were also significantly higher (p < 0.01) in the IGF-1 (89.3%, 28.5%, and 20.6%) and lycopene (84.2%, 30.8%, and 32.7%) groups than in the control (75.1%, 20.3%, and 12.2%) and α-tocopherol (76.7%, 23.2%, and 14.4%) groups. Lycopene produced the highest blastocyst yield. Mitochondrial fluorescence intensity was significantly greater (p < 0.01) in all supplemented groups compared with the control. Diffuse mitochondrial distribution predominated in IGF-1- and lycopene-treated oocytes, indicating improved cytoplasmic competence and metabolic activity, whereas α-tocopherol treatment was associated with increased peripheral mitochondrial localization.

**Conclusion:**

Supplementation of IVM medium with 100 ng/mL IGF-1 or 0.2 μM lycopene significantly enhances nuclear maturation, mitochondrial activity, and embryo developmental competence of buffalo oocytes. Lycopene demonstrated the most pronounced improvement in blastocyst formation, suggesting superior antioxidant protection during maturation. These findings highlight the importance of optimizing mitochondrial function and oxidative balance in buffalo IVEP systems and provide a potential strategy to improve reproductive biotechnology outcomes in buffalo.

## INTRODUCTION

Buffaloes (*Bubalus bubalis*) represent a major component of livestock production systems in many Mediterranean and South Asian countries. Despite their considerable economic and agricultural importance, the reproductive efficiency of buffalo populations has declined in recent decades. This decline is largely attributed to inadequate genetic selection programs, poor reproductive management, and suboptimal nutritional practices [1]. To overcome these limitations and accelerate genetic improvement, assisted reproductive technologies have increasingly been adopted in buffalo breeding programs.

Among these technologies, *in vitro* embryo production (IVEP) has emerged as a powerful tool for the rapid multiplication of genetically superior animals. The IVEP system generally involves three sequential stages: *in vitro* maturation (IVM) of oocytes, *in vitro* fertilization (IVF), and *in vitro* culture (IVC) of embryos [2]. Among these stages, the maturation of oocytes under *in vitro* conditions represents one of the most critical determinants of embryo developmental competence. However, the efficiency of buffalo IVEP systems remains relatively low compared with that of cattle. One of the principal factors limiting oocyte competence during *in vitro* maturation (IVM) is oxidative stress, which arises from an imbalance between the production and neutralization of reactive oxygen species (ROS) within the culture environment [3].

To mitigate oxidative stress and improve oocyte quality, various supplements have been incorporated into IVM media. These supplements may include hormones, growth factors, and antioxidants that modulate cellular metabolism and protect oocytes from oxidative damage. Growth factors such as insulin-like growth factor-1 (IGF-1) and antioxidants such as lycopene and α-tocopherol have attracted considerable attention because of their ability to enhance oocyte maturation, fertilization success, and embryo developmental competence. Nevertheless, most previous studies have evaluated these supplements individually. Investigations involving IGF-1 have primarily been conducted in buffalo and bovine species, whereas lycopene supplementation has mainly been studied in cattle and pigs. Similarly, α-tocopherol has been examined primarily in cattle, sheep, and porcine models. Consequently, comparative evaluations of these supplements under identical IVC conditions in buffalo oocytes remain scarce.

The addition of growth factors and antioxidants to embryo culture media has been shown to enhance blastocyst development, increase hatching rates, and improve pregnancy outcomes in several livestock species [4]. Among these supplements, IGF-1 is considered one of the most important growth factors involved in ovarian follicular development and early embryogenesis. IGF-1 acts as a potent anti-apoptotic factor during preimplantation embryo development in buffalo (*Bubalus bubalis*) [5]. Supplementation of maturation media with 50 ng/mL IGF-1 has been reported to improve nuclear maturation rates of buffalo oocytes [6], whereas higher concentrations (100 ng/mL) significantly enhance maturation, cleavage, morula formation, and blastocyst development [7]. IGF-1 promotes cell survival, proliferation, and steroidogenesis in granulosa cells (GCs) and plays a crucial role in regulating follicular growth, oocyte maturation, and embryo development [8].

Lycopene is another promising supplement for improving oocyte competence. It is a naturally occurring carotenoid characterized by a highly conjugated molecular structure consisting of 13 double bonds, 11 of which are conjugated. This structure confers exceptionally strong antioxidant properties, making lycopene approximately twice as effective as β-carotene in scavenging free radicals [9, 10]. Lycopene has been shown to enhance mitochondrial activity, reduce apoptosis, and increase both the inner cell mass (ICM) and total cell number in bovine blastocysts [11–13]. Residiwati *et al*. [14] demonstrated that supplementation with 0.2 μM lycopene significantly improves nuclear maturation, fertilization rates, cleavage, and blastocyst development in bovine oocytes cultured under non-stress conditions.

Another important antioxidant used in reproductive biotechnology is α-tocopherol, the biologically active form of vitamin E. As a lipid-soluble antioxidant, α-tocopherol protects cellular membranes from oxidative damage by scavenging free radicals and preventing lipid peroxidation [15]. A previous study has reported that supplementation of buffalo oocytes with 100 μM vitamin E during IVM increases maturation, cleavage, morula, and blastocyst rates [16]. In bovine embryos, α-tocopherol has also been shown to improve embryonic quality during IVM [17]. Furthermore, α-tocopherol supplementation enhances granulosa cell viability in pigs [18], supports folliculogenesis and oocyte competence in cattle [15], and improves embryonic development in gilts [19] and sheep [20]. Vitamin E also prevents membrane damage [21], inhibits lipid peroxidation in bovine cells [22], and reduces apoptosis in mammalian oocytes [23].

Mitochondria play a fundamental role in regulating oocyte maturation and embryonic development. These organelles serve as the primary source of cellular energy in the form of adenosine triphosphate (ATP), which is essential for transcriptional and translational processes occurring during oocyte maturation [24]. During maturation, mitochondrial DNA copy number increases progressively, and mitochondrial distribution patterns within the oocyte cytoplasm undergo substantial changes [24]. The proper distribution and activity of mitochondria are therefore essential for maintaining oocyte developmental competence.

Growth factors and antioxidants are known to modulate mitochondrial function in *in vitro*-matured oocytes. For example, IGF-1 has been reported to enhance mitochondrial polarization, stimulate ATP production by activating the PI3K/Akt-signaling pathway, and promote steroid biosynthesis, thereby facilitating the transition from the immature to the mature stage [25]. Similarly, antioxidants can improve mitochondrial performance by reducing reactive oxygen species (ROS) levels and protecting mitochondrial membranes from oxidative damage. Lycopene has been shown to increase mitochondrial activity, reduce ROS accumulation, and decrease apoptosis in animal oocytes [26]. Likewise, α-tocopherol stabilizes membrane-bound lipids and protects mitochondria against oxidative stress caused by ROS [27].

These protective mechanisms ultimately lead to improved IVM rates and enhanced embryo developmental competence in several livestock species [28]. Mature metaphase II (MII) oocytes typically contain approximately 500,000 copies of mitochondrial DNA, which are essential for maintaining mitochondrial activity and regulating intracellular oxidative balance [29]. The spatial distribution of mitochondria within the oocyte cytoplasm is tightly regulated and plays a crucial role in cellular organization and metabolic activity [30].

Recent studies have highlighted the importance of mitochondria-targeted antioxidants, which can accumulate within mitochondria at concentrations 100–1,000 times higher than those found in the surrounding cytoplasm [31]. Monitoring mitochondrial distribution patterns has therefore emerged as a valuable biological indicator for assessing oocyte quality and developmental competence [32]. During meiosis I (MI), mitochondria gradually surround the developing spindle apparatus and form clusters within the cytoplasm. By the MII stage, mitochondria become widely dispersed throughout the cytoplasm, with higher concentrations observed in the spindle hemisphere and cortical regions of the oocyte [33]. Nevertheless, considerable variability exists in the intensity and distribution of mitochondria among oocytes of different species and developmental stages.

Despite increasing recognition of the importance of mitochondrial dynamics in oocyte maturation, a comprehensive characterization of mitochondrial distribution patterns during buffalo oocyte maturation remains limited. Moreover, objective and quantitative approaches to evaluating mitochondrial distribution in oocytes remain underdeveloped [32].

Despite substantial advances in reproductive biotechnology, the efficiency of buffalo *in vitro* embryo production systems remains significantly lower than that observed in cattle. Although numerous studies have examined the effects of individual supplements such as IGF-1, lycopene, or α-tocopherol on oocyte maturation and embryo development, most investigations have evaluated these compounds independently and under different experimental conditions. Consequently, the relative efficacy of these supplements and their specific influence on mitochondrial dynamics in buffalo oocytes remain poorly understood. Furthermore, limited information is available regarding the relationship between mitochondrial distribution patterns, mitochondrial activity, and subsequent embryo developmental competence in buffalo. A comprehensive comparative evaluation of growth factors and antioxidants under identical IVM conditions is therefore required to elucidate their mechanisms of action and identify optimal strategies to improve buffalo IVEP outcomes.

Therefore, the present study aimed to compare the effects of IGF-1, lycopene, and α-tocopherol supplementation during IVM on the developmental competence of buffalo (*Bubalus bubalis*) oocytes. Specifically, this study sought to: (i) assess the influence of these supplements on nuclear maturation and cytoplasmic maturation of buffalo oocytes; (ii) determine their effects on embryo developmental parameters, including cleavage, morula formation, and blastocyst development; and (iii) investigate changes in mitochondrial activity and distribution patterns using confocal microscopy. By integrating reproductive outcomes with mitochondrial dynamics, this study provides a mechanistic framework for understanding how growth factors and antioxidants influence oocyte quality and embryo development in buffalo IVEP systems.

## MATERIALS AND METHODS

### Ethical approval

All procedures involving animal biological materials were performed in strict accordance with national and international guidelines for the ethical use of animals in research. This study did not involve any live experimental animals; ovaries were sourced exclusively from routinely slaughtered adult buffaloes (*Bubalus bubalis*) at the El-Sharkawy Governmental Abattoir, Qalyubia, Egypt, which operates under the Ministry of Agriculture and complies with national animal welfare and food safety regulations. The experimental protocol, including ovaries and oocyte collection, transport, handling, and laboratory processing, was reviewed and approved by the Ethics Committee of the National Research Center (NRC), Cairo, Egypt, under approval number NRC, ID: 19/145. The committee confirmed that no live animals were subjected to experimental manipulation, restraint, hormonal treatment, or invasive procedures. No discomfort, pain, or distress was inflicted on animals, as all tissues were collected postmortem from animals slaughtered for routine meat production. Biosafety and biosecurity procedures were followed throughout the sample handling process, including the use of sterile containers, leak-proof packaging, and adherence to BSL-2 standards within the NRC Embryo and Genetic Resources Conservation Laboratory. Ovaries were transported in sterile 0.9% saline supplemented with antibiotics at 30°C–35°C, ensuring that tissue handling complied with ethical and veterinary-health standards. All personnel involved in sample collection and laboratory procedures were trained and certified in animal tissue handling and laboratory biosafety. This work adheres to the guidelines of: The International Council for Laboratory Animal Science, The OIE/WOAH Code of Practice for the Use of Animals in Research, ARRIVE 2.0 recommendations (where applicable to studies using animal biological material), and National Ethical Guidelines for Animal Use in Research issued by the Egyptian Ministry of Higher Education and Scientific Research. Because no live animals were used and no experimental treatments were applied to the donor animals, the study is considered to involve minimal ethical risk.

### Study period and location

The study was conducted during the buffalo breeding season, from October to March, between 2022 and 2025. The *in vitro* development of embryos and mitochondrial function in buffalo oocytes were conducted at the Embryo and Genetic Resources Conservation Bank at the National Research Centre, Cairo, Egypt.

### Collection and transport of the buffalo ovaries

Ovaries were collected from clinically healthy, non-pregnant adult buffaloes (*Bubalus bubalis*) aged 2–6 years immediately after slaughter at the El-Sharkawy abattoir in Qalyubia, Egypt, between 2022 and 2025 during the breeding season (October–March). Ovaries were transported to the laboratory in a thermos flask containing sterile normal saline solution (NSS, 0.9% NaCl) maintained at 32°C–35°C and supplemented with 100 μg/mL of streptomycin and 100 IU of penicillin within 2–3 h post-slaughter. A total of 1,876 ovaries were processed in the laboratory. Each ovary was washed repeatedly in normal saline solution at 37°C [34].

### Retrieval and grading of buffalo oocytes

#### Follicular aspiration

Cumulus–oocyte complexes (COCs, n = 4,250 oocytes) were aspirated from 2–8 mm follicles, and only excellent and good quality oocytes were selected, whereas fair and denuded (n = 1,342 oocytes) were excluded. COCs were retrieved from follicles that ranged in diameter from 2 to 8 mm using a sterile disposable 20 mL syringe with an 18-gauge needle containing 1 mL of phosphate-buffered saline (PBS) supplemented with 6 mg/mL of bovine serum albumin (fraction V) and 50 μg/mL of gentamicin. After aspiration, the follicular fluid was placed in a 15-mL sterile Falcon tube and allowed to settle in a water bath at 37°C for 15 min.

#### Washing and handling

The COCs were washed three times in the aspiration medium to remove blood and debris. Handling was performed carefully to preserve oocyte integrity.

#### Grading criteria

The quality of the COCs was examined using a stereomicroscope (Zeiss, Oberkochen, Germany) at 90× magnification [35]. The quality of the oocytes was classified into four categories based on the condition of the cumulus layers and the homogeneity of the cytoplasm: excellent, good, fair, and denuded [36]. Excellent: Oocytes with more than five layers of cumulus cells and dark cytoplasm with uniform granulation. Good: Dark cytoplasm with uniform granulation and three to five layers of cumulus cells. Fair: Oocytes that are partially enclosed by cumulus cells, with minimally granulated cytoplasm. Denuded: Oocytes that lack cumulus cells and are only covered by zona pellucida [36, 37]. Only excellent and good quality oocytes (n = 2908) were selected for subsequent experiments, whereas fair and denuded oocytes (n = 1342) were excluded. Excellent oocytes = 1650 (38.82%), good oocytes = 1,258 (29.6%), fair oocytes = 770 (18.12%), and denuded oocytes = 572 (13.46%).

### IVM of buffalo oocytes

#### Composition of the maturation medium

The basic maturation medium consisted of TCM-199 (M4530, Sigma-Aldrich, St. Louis, MO, USA) with 10% fetal calf serum (FCS) + 10 μg/mL follicle-stimulating hormone (FSH) (Follitropin-V, Bioniche Animal Health, Belleville, ON, Canada) + 50 μg/mL gentamicin and filtered using 0.2 μm syringe filter (Thermo Fisher Scientific, Waltham, MA, USA) and incubated for at least 2 h in a humidified atmosphere (95%) under 5% CO_2_ at 38.5°C before culturing of the oocytes. Excellent and good oocytes *in vitro* were matured in 500 μL maturation medium for 22 h in a humidified atmosphere (95%) under 5% CO_2_ at 38.5°C.

#### Preparation of IGF-1, lycopene, and α-tocopherol

1. The dose and preparation of IGF-1 are according to Ismail *et al*. [7]
2. The dose and preparation of lycopene were performed according to Residiwati *et al*. [14]. To prepare the stock solution of lycopene, 1 mg of lycopene (molar mass: 536.873; SMB00706, CAS number 502-65-8, Sigma-Aldrich, St. Louis, MO, USA) was dissolved in 5 μL of dimethyl sulfoxide, and then 1 mL of distilled water was added. Aliquots of 20 μL of lycopene stock were prepared and stored at −80 °C. To prepare the lycopene working solution on the day of maturation, 10 μL of lycopene stock solution was added to 1990 μL of distilled water. After that, the lycopene working solution was added into each well (1 μL of working solution into 500 μL of maturation medium), resulting in 0.2 μM lycopene and protected from light.
3. The dose and preparation of α-tocopherol were as described by Thiyagarajan and Valivittan [16]. Preparation of vit E as a molar mass (430.7) g/L and vit E as a fat-soluble stock solution, each 1 mL contain 0.95 g; addition of 1 μL of vit E to 219 μL of basic maturation media for a concentration of 10000 μM, then final working solution is 10 μL from 220 and added to 990 mL maturation media for a concentration of 100 μM.

#### Culture conditions

*In vitro* buffalo oocyte maturation was performed in a humidified CO_2_ incubator (Binder, Tuttlingen, Germany) with 5% CO_2_ at 38.5°C for 22 h. This experiment was conducted with 10 replicates for each group to ensure the reliability of the results.

#### Assessment of cytoplasmic maturation

Cytoplasmic maturation was assessed in all *in vitro*-matured buffalo oocytes based on the extent of cumulus cell expansion, which was classified into four grades [3]. G0: No expansion of cumulus cells. GI: Oocytes exhibiting slight expansion of the cumulus cell outer layer. GII: Oocytes with moderet expaned cumulus cells. GIII: Oocytes with fully expanded cumulus cells.

#### Assessment of nuclear maturation

Nuclear maturation evaluation was based on the presence of the first polar body (1^st^ PB) in the oocyte, which was determined after gently removing the cumulus cells of all *in vitro*-matured buffalo oocytes through pipetting at the end of the maturation phase. An inverted microscope with 200× magnification (Zeiss) was used to detect the 1^st^ PB.

### *In vitro* fertilization (IVF) of buffalo oocytes

#### Semen thawing and washing

Frozen buffalo semen was purchased from the Abbasia Artificial Insemination Center, General Organization for Veterinary Services, Ministry of Agriculture, Egypt. The same batch of the same bull with proven fertility was used throughout the study (Egy. Buff. ASWAD 656 ABASSIA AIC). A 0.25 mL straw of frozen semen was thawed in a water bath at 37°C for 30 s. The spermatozoa (60%–70% progressive motility) were washed with 3 mL of sperm Tyrode’s albumin lactate pyruvate medium supplemented with 1 μg/mL heparin, 3 mg/mL BSA, 2.5 mg/mL hypotaurine and 50 μg/mL gentamicin. The mixture was then centrifuged at 1,800 rpm (approximately 700 × *g*) for 10 min. After centrifugation, the semen pellet was resuspended in 3 mL of Fert-TALP medium and centrifuged again at 1,800 rpm (approximately 700 × *g*) for 5 min.

#### Sperm concentration adjustment

Following these two centrifugation steps, the remaining semen pellet was combined with 200 μL Fert-TALP medium, and the sperm number was counted using a hemocytometer. The final sperm concentration was modified to 1 × 10^6^ spermatozoa/mL before being placed in the four-well plate.

#### Insemination procedure

Sperm suspension with 300 μL Fert-TALP medium was placed into a four-well culture plate and covered with 200 μL of warm mineral oil. Both oocytes and sperm were cultured in a CO_2_ incubator with a 5% CO_2_ humidified atmosphere at 38.5°C for 18 h.

#### Fertilization assessment

Fertilization was assessed based on the presence of the second PB. The fertilization rate was evaluated according to established guidelines [3]. Polyspermy-fertilized oocytes were excluded from assessment and IVC for development.

### IVC of buffalo oocytes

#### Culture medium composition

Modified synthetic oviduct fluid medium (mSOF) supplemented with 5 mg/mL BSA and 50 μg/mL gentamicin. Fertilized oocytes were cultured in a four-well culture plate using mSOF medium (50 oocytes/500 μL medium) for 7 days (change medium every 2 days) in a humidified atmosphere (95%) under 5% CO_2_ at 38.5°C. A fresh culture medium was introduced every two days for a total of seven days.

#### Embryo staging and evaluation

Cleavage and embryo development rates were assessed on days 2, 5, and 7 using an inverted microscope (Zeiss). The embryo cleavage, morula, and early blastocyst rates were evaluated according to *El-Sanea et al*. [3].

### Mitochondrial staining and confocal microscopy: Fixation and permeabilization

The *in vitro*-matured buffalo oocytes from all groups, after 22 h of decumulation (removal of the cumulus cells), were fixed in 4% paraformaldehyde for 24 h to preserve their spherical shape. The cells were then rinsed with PBS containing 0.1% polyvinylpyrrolidone, followed by PBS containing Triton X-100 (0.2%) to enhance the cell membrane permeability for staining.

#### The staining procedure

Mito Tracker Red FM stain (Thermo Fisher Scientific), a fluorescent marker specific to mitochondria, was used to stain mature buffalo oocytes *in vitro*. Moreover, 4′,6-diamidino-2-phenylindole (DAPI), a nuclear counterstain for mature oocytes, was used to detect nuclear DNA and the first PB. The fixed and washed *in vitro*-matured buffalo oocytes were rinsed in phosphate-buffered saline (PBS) with 500 nM Mito Tracker Red FM for approximately 30 min at 37°C in a CO_2_ incubator. After two washes with PBS/PVP, the stained buffalo oocytes were incubated in PBS containing 5 μg/mL DAPI. Oocytes were washed twice in PBS/PVP. After a final wash in phosphate-buffered saline, the stained buffalo oocytes were visualized in a 12-mm diameter glass-bottom culture plate (Thermo Fisher Scientific) using a confocal microscope (Zeiss LSM 710). All the steps were done away from direct light, and were covered with aluminum foil to keep the fluorescence of the stained oocytes.

#### Imaging parameters

A confocal microscope with four laser beams (Zeiss LSM 710) and Zen 2010 software (https://www.zeiss. com/microscopy/us/products/software/zeiss-zen-lite.html) were used to image the stained oocytes. A confocal microscope connected to a P-TMP unit was used to take images without fluorescence; then the red and blue laser beams were used to image red mitochondria and blue for nuclear DNA and the 1st PB. The confocal microscope was calibrated using the standard stained slide that came with the microscope to ensure the laser beams were functioning properly before imaging. We selected the black oocytes and white under 200× magnification. Then, the laser beams were used to capture images of the oocytes. The appearance of the nucleus and the first polar body (PB), which should be stained with DAPI to show blue fluorescence, along with the red image of the mitochondria, are the key indicators for imaging. The DAPI stain was examined using excitation and emission wavelengths of 358 and 460 nm, respectively. The Mito Tracker Red stain was analyzed using an emission filter set to 644 nm and an excitation wavelength of 581 nm. An optical section was captured at the plane where the nucleus was visible for each oocyte.

#### Quantification and distribution criteria

A confocal microscope (Zeiss LSM 710) was used to assess the mitochondrial distribution and morphology of the stained oocytes. The distribution of mitochondria in oocytes was categorized as follows: (1) peripheral distribution, where mitochondria were found on the inner surface of the zona pellucida; (2) semi-peripheral distribution, where mitochondria were found in the inner region of the oocyte but were distributed unevenly; (3) diffuse distribution, characterized by a homogeneous arrangement of mitochondria throughout the entire oocyte; and (4) semi-diffused distribution, where mitochondria were concentrated in the center with an uneven distribution toward the oocyte wall. The confocal microscope’s software automatically detected mitochondrial intensity [38]. Each group of *in vitro*-matured oocytes underwent three replicates of this experiment, with each replicate containing 10 oocytes.

### Experimental design and replication

**Figure 1:**
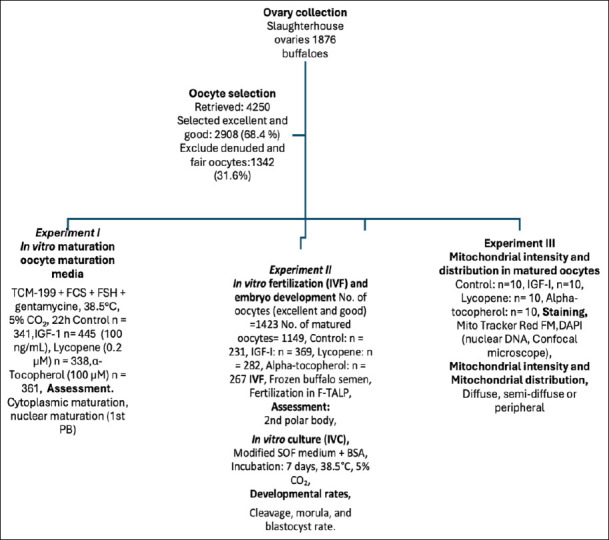
Schematic overview of the experimental workflow for *in vitro* maturation, fertilization, embryo development, and mitochondrial analysis in buffalo oocytes with growth factor and antioxidant supplementation (IGF-1, lycopene, and α-tocopherol).

The excellent and good oocytes were randomly allocated during IVM and culture, 50 oocytes in 500 μL media/well of a four-well tissue culture plate. The oocytes were randomly distributed into four experimental groups: control (no supplementation), IGF-1 (100 ng/mL), lycopene (0.2 μM), and α-tocopherol (100 μM). Each experiment was replicated ten times using independent oocyte batches. Oocytes were randomly allocated into the control, IGF-1, lycopene, and α-tocopherol groups. Each experiment was replicated 10 times. The sample sizes were based on power analysis to detect significant differences (power = 0.8, α = 0.05). Experiments were conducted on separate days, and Figure 1 summarizes the number of replicates and oocytes analyzed per group.

#### Experiment I: Effects of IGF-1, lycopene, and α-tocopherol on IVM of buffalo oocytes

Excellent and good oocytes (n = 1485) were matured *in vitro* in maturation media in a four-well culture dish (50 oocytes in 500 μL media/well) in four different groups. (1) TCM-199 was used as a control group (n = 341), (2) TCM-199 + 100 ng/mL IGF-1 (n = 445), (3) TCM-199 + 0.2 μM lycopene (n = 338), and (4) TCM-199 + 100 μM α-tocopherol (n = 361) in a humidified CO_2_ incubator (Binder, Tuttlingen, Germany) with 5% CO_2_ at 38.5°C for 22 h while fair and denuded oocytes were excluded. This experiment was conducted with 10 replicates for each group to ensure the reliability of the results.

#### Experiment II: Effect of IGF-1, lycopene, and α-tocopherol on buffalo oocyte development

After the *in vitro* oocyte maturation phase as described in Experiment I, four groups of matured oocytes (n = 1149), the control group (n = 231), the IGF-1 group (n = 369), the lycopene group (n = 282), and the α-tocopherol group (n = 267) were cultured as 50 oocytes/500 μL of Fert-TALP in a four-well culture dish. Both the oocytes and sperms were cultured in a CO_2_ incubator with a 5% CO_2_ humidified atmosphere at 38.5°C for 18 h. The presumptive zygotes were then cultured in modified synthetic oviductal fluid (mSOF) medium supplemented with 5 mg/mL BSA and 50 μg/mL gentamicin and incubated for 7 days in 5% CO_2_ at 38.5°C. Cleaved oocytes and developing embryos were examined on days 2, 5, and 7. A fresh culture medium was introduced every two days. Fertilization, cleavage, morula formation, and blastocyst formation rates were evaluated. Each group in this experiment consisted of 10 replicates.

#### Experiment III: Effect of IGF-1, lycopene, and α-tocopherol on intensity and distribution in in vitro-matured buffalo oocytes

The four groups of *in vitro*-matured buffalo oocytes (n = 120), each group of *in vitro*-matured oocytes underwent three replicates of this experiment, with each replicate containing ten oocytes, were used for subsequent experiments for staining using Mito Tracker and DAPI stain for the detection of the viability and mitochondrial intensity, and distribution as described above.

### Statistical analysis

Statistical analysis was performed using the SPSS version 28.0 software (SPSS Inc., Chicago, IL, USA). Data were tested for normality before analysis. Quantitative data are expressed as mean ± standard error. Differences among groups were evaluated using one-way analysis of variance followed by Tukey’s post hoc test, while proportional data were analyzed using the chi-square test or Fisher’s exact test [39]. Differences were considered statistically significant at p ≤ 0.05.

## RESULTS

### Experiment I: Effect of IGF-1, lycopene, and α-tocopherol on IVM rates of buffalo oocytes

#### Cumulus cell expansion rate

**Figure 2:**
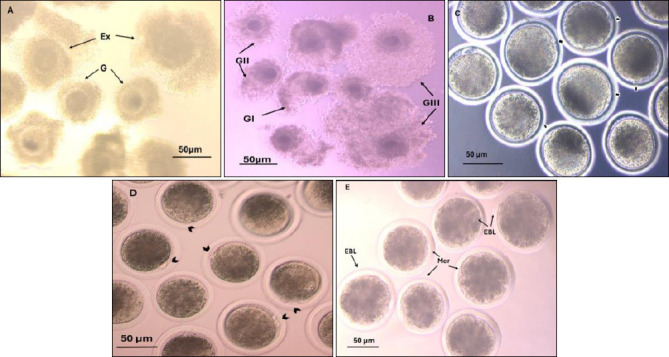
Buffalo oocyte quality and oocyte developmental competence. A) Oocyte quality (G=good, Ex = excellent). B) Cumulus expansion (GIII =full expansion, GII = moderate expansion, GI = little expansion). C) Nuclear matured oocytes, the arrow shows the 1st polar body. D) Fertilized oocytes, the arrow shows the 2nd polar body. F) Transferable embryo (Mor = morula, EBL = early blastocyst). These pictures were taken using a Zeiss inverted microscope at 200× magnification, with a scale of 50 μm.

The effect of the addition of 100 ng/mL IGF-1 (IGF-1 group), 0.2 μM lycopene (lycopene group), and 100 μM α-tocopherol (α-tocopherol group) to IVM medium (TCM-199) on cumulus cell expansion of *in vitro*-matured excellent and good (Figure 2A) buffalo oocytes is demonstrated in this study (Table 1, Figure 2B) showing that the lycopene group significantly (p < 0.01) increased the percentage (mean ± S. E) of oocytes with Grade III and GII cumulus cell expansion (58 ± 0.46%, 28.63 ± 0.49% respectively) when compared to TCM group (41.34 ± 0.46%, 16.76 ± 0.40% respectively) or those that were matured in IGF-1 group (41.77 ± 0.32%,16.82 ± 0.3%, respectively) and α-tocopherol Group (42.57 ±0.82%, 17.62±0.27%, respectively). On the other hand, the Gl and G0 cumulus expansion of oocytes matured with the lycopene group was significantly lower (p < 0.01) (8.44 ± 0.62%, 5.04 ± 0.39% respectively) than the TCM group (16.81 ± 0.73%, 25.07 ± 0.52% respectively) or the IGF-1 (20.01 ± 0.32%, 21.42 ± 0.30% respectively) and α-tocopherol. Groups (20.06 ± 0.37%,19.75 ± 0.89% respectively). The effect of the addition of 100 ng/mL IGF-1 (IGF-1 group), 0.2 μM lycopene (lycopene group), and 100 μM α-tocopherol (α-tocopherol group) to the IVM medium (TCM-199) on the cumulus cell expansion of *in vitro*-matured buffalo oocytes is demonstrated in this study (Table 1, Figure 2B) showed that the lycopene group significantly (p < 0.01) increased the percentage of oocytes with Grade III and GII cumulus cell expansion (58% and 28.7%, respectively) when compared to the TCM group (41.3% and 16.7%, respectively) or those that were matured in the IGF-1 group (41.8% and 16.9%, respectively) and α-tocopherol Group (42.9% and 17.7%, respectively). On the other hand, the Gl and G0 cumulus expansion of oocytes matured with the lycopene group was significantly lower (p < 0.01) (8.3% and 5%, respectively) than that in the TCM group (16.7% and 25.2%, respectively) or the IGF-1 (20% and 21.3%, respectively) and α-tocopherol Groups (19.9% and 19.4%, respectively).

**Table 1:** Effect of IGF-1, lycopene, and α-tocopherol supplementation on cumulus cell expansion (mean ± standard error and percentage) of *in vitro*-matured buffalo oocytes.

Groups	Total oocytes	GIII (No.)	GIII (Mean ± SE)	GIII (%)	GII (No.)	GII (Mean ± SE)	GII (%)	GI (No.)	GI (Mean ± SE)	GI (%)	G0 (No.)	G0 (Mean ± SE)	G0 (%)
Control	341	141	41.34 ± 0.46^b^	41.3^b^	57	16.76 ± 0.40^b^	16.7^b^	57	16.81 ± 0.73^b^	16.7^b^	86	25.07 ± 0.52^a^	25.2^a^
IGF-1	445	186	41.77 ± 0.32^b^	41.8^b^	75	16.82 ± 0.30^b^	16.9^b^	89	20.01 ± 0.32^a^	20.0^a^	95	21.42 ± 0.30^b^	21.3^b^
Lycopene	338	196	58.00 ± 0.46^a^	58.0^a^	97	28.63 ± 0.49^a^	28.7^a^	28	8.44 ± 0.62^c^	8.3^c^	17	5.04 ± 0.39^c^	5.0^c^
α-tocopherol	361	155	42.57 ± 0.82^b^	42.9^b^	64	17.62 ± 0.27^b^	17.7^b^	72	20.06 ± 0.37^a^	19.9^a^	70	19.75 ± 0.89^b^	19.4^b^

Replicates = 10 a,b,c Means within the same column bearing different superscripts differ significantly (p < 0.01). IGF-1 = insulin-like growth factor-1, ng = nanogram, μM = micromolar, SE = Standard error.

#### Nuclear maturation rate

Mature oocytes were examined for the presence of first Pb as an indication for meiosis II (Table 2, Figure 2C) and found that the maturation rate (mean ± SE) in the IGF-1 group and lycopene group (85.12 ± 0.46%, 87.07 ± 1.41%, respectively) was significantly (p < 0.01) higher than the TCM (73.34 ± 0.16%) or α-tocopherol group (76.27±0.78 %). The *in vitro*-matured buffalo oocytes without a PB in the TCM and α-tocopherol groups showed a significant (p < 0.01) difference (26.66 ± 0.16%, 23.73 ± 0.78%, respectively) compared with the IGF-1 and lycopene groups (14.89 ± 0.46%, 12.93 ± 1.41%, respectively). Mature oocytes were examined for the presence of first Pb as an indication for meiosis II (Table 2, Figure 2C). The maturation rate in the IGF-1 and lycopene groups (85.2% and 87.30%, respectively) was significantly (p < 0.01) higher than that in the TCM (73.3%) or α-tocopherol group (76.20%). The *in vitro*-matured buffalo oocytes without a PB in the TCM and α-tocopherol groups showed a significant (p < 0.01) difference (26.7% and 23.80%, respectively) compared with the IGF-1 and lycopene groups (14.8% and 12.70%, respectively).

**Table 2:** Effect of IGF-1, lycopene, and α-tocopherol supplementation on nuclear maturation rate (mean ± standard error and percentage) of *in vitro*-matured buffalo oocytes.

Groups	Total oocytes*	Maturation rate (1st PB)			Oocytes without a polar body rate		

		No.	Mean ± SE (%)	%	No.	Mean ± SE (%)	%
Control	341	250	73.34 ± 0.16^b^	73.3^b^	91	26.66 ± 0.16^a^	26.7^a^
IGF-1	445	379	85.12 ± 0.46^a^	85.2^a^	66	14.89 ± 0.46^b^	14.8^b^
Lycopene	338	295	87.07 ± 1.41^a^	87.3^a^	43	12.93 ± 1.41^b^	12.7^b^
α-Tocopherol	361	275	76.27 ± 0.78^b^	76.2^b^	86	23.73 ± 0.78^a^	23.8^a^

Replicates = 10

* Total number of oocytes: number of excellent and good quality oocytes. a,b Means within the same column bearing different superscripts differ significantly (p < 0.01). IGF-1 = insulin-like growth factor-1, PB = polar body, ng = nanogram, μM = micromolar, SE = Standard error.

### Experiment II: Effect of IGF-1, lycopene, and α-tocopherol on embryo development in buffalo

The results showed no significant difference (p > 0.05) between control, IGF-1, lycopene, and α-tocopherol groups (85.37 ± 0.61%, 88.03 ± 0.75%, 87.82±1.22%, and 87.99 ± 1.01%, respectively) in the fertilization rate (mean ± S. E). Cleavage rate, morula rate, and blastocyst rate (mean ± S. E) were significantly (p < 0.01) higher in the IGF-1 group (88.99 ± 0.57%, 28.52 ± 1.03% and 20.70 ± 0.42%, respectively) and lycopene group (84.33 ± 0.33%, 30.91 ± 0.84%, and 32.71 ± 0.36%, respectively) when compared with TCM group (75.11 ± 0.64%, 20.37 ± 0.48% and 12.31 ± 0.80%, respectively) and α-tocopherol group (76.51 ± 0.45%, 22.85 ± 0.73% and 14.28 ± 0.85%, respectively). The blastocyst rate (mean ± S. E) was significantly higher (p < 0.01) in the lycopene group than in the IGF-1 group (Table 3, Figure 2D and E). The results showed no significant difference (p > 0.05) in fertilization rate among the control, IGF-1, lycopene, and α-tocopherol groups (85.3%, 88.3%, 87.6%, and 88.4%, respectively). Cleavage rate and morula rate and blastocyst rate were significantly (p < 0.01) higher in the IGF-1 group (89.3%, 28.5%, and 20.6%, respectively) and lycopene group (84.2%, 30.8%, and 32.7%, respectively) than in the TCM group (75.10%, 20.3%, and 12.2%, respectively) and α-tocopherol group (76.7%,23.2% and 14.4% respectively). The blastocyst rate was significantly higher (p < 0.01) in the lycopene group than in the IGF-1 group (Table 3, Figures 2D and E).

**Table 3:** Effect of IGF-1, lycopene, and α-tocopherol supplementation on embryo development (mean ± standard error and percentage) in buffalo.

Groups	No. of oocytes*	Fertilization rate			Cleavage rate			Morula rate			Blastocyst rate		

		No.	Mean ± SE (%)	%	No.	Mean ± SE (%)	%	No.	Mean ± SE (%)	%	No.	Mean ± SE (%)	%
Control	231	197	85.37 ± 0.61^a^	85.3^a^	148	75.11 ± 0.64^b^	75.1^b^	30	20.37 ± 0.48^b^	20.3^b^	18	12.31 ± 0.80^c^	12.2^c^
IGF-1	369	326	88.03 ± 0.75^a^	88.3^a^	291	88.99 ± 0.57^a^	89.3^a^	83	28.52 ± 1.03^a^	28.5^a^	60	20.70 ± 0.42^b^	20.6^b^
Lycopene	282	247	87.82 ± 1.22^a^	87.6^a^	208	84.33 ± 0.33^a^	84.2^a^	64	30.91 ± 0.84^a^	30.8^a^	68	32.71 ± 0.36^a^	32.7^a^
α-tocopherol	267	236	87.99 ± 1.01^a^	88.4^a^	181	76.51 ± 0.45^b^	76.7^b^	42	22.85 ± 0.73^b^	23.2^b^	26	14.28 ± 0.85^c^	14.4^c^

Replicates = 10

* Oocytes with first polar body. a,b,c Means within the same column bearing different superscripts differ significantly (p < 0.01). IGF-1 = insulin-like growth factor-1, PB = polar body, ng = nanogram, μM = micromolar.

### Experiment III: Effect of IGF-1, lycopene, and α-tocopherol on mitochondrial intensity and distribution

**Figure 3:**
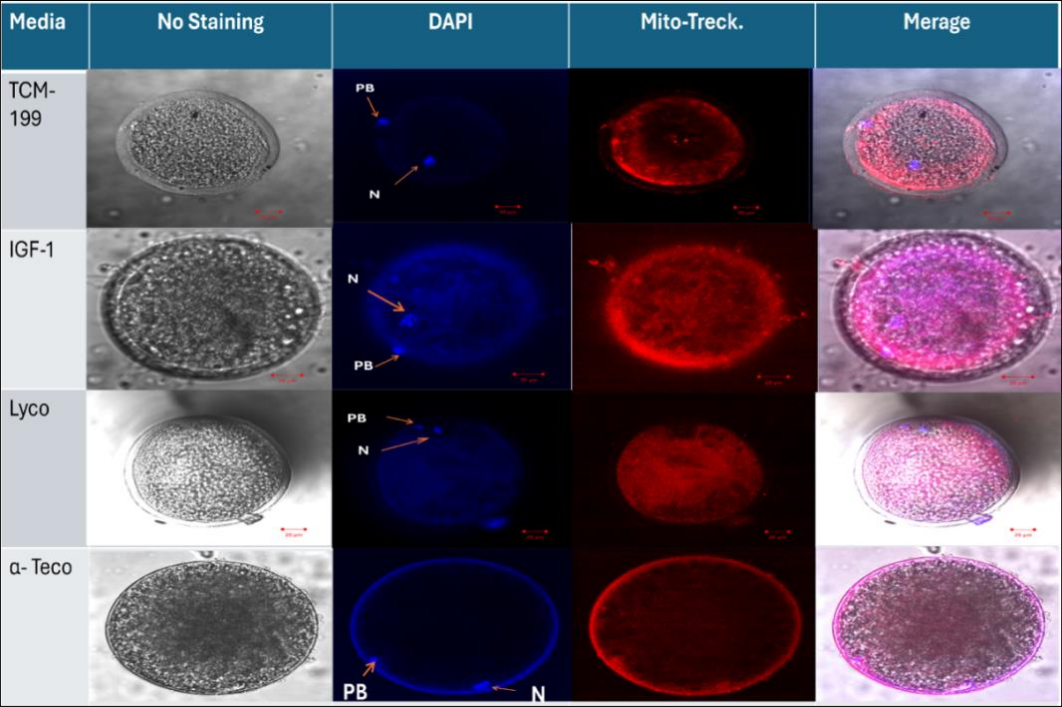
Detection of the effects of IGF-1, lycopene, and α-tocopherol on viability and mitochondrial distribution of *in vitro*-matured buffalo oocytes at 200X magnification, scale 20 μm using a confocal microscope (Zeiss LSM 710). The viability of mature oocytes appears in the extrusion of the 1st Pb. Mature oocytes without staining; matured oocytes stained with DAPI stain; N = nucleus; Pb = polar body; TCM media (control) showed a semi-diffused distribution, while the IGF-1 and lycopene groups showed a diffused distribution, and the α-tocopherol group showed a peripheral distribution of *in vitro*-matured oocytes stained with Mito Tracker stain.

Mitochondrial mean ± S. E intensity (Table 4) was significantly (p < 0.01) higher in the IGF-1 group (290.72 ± 10.19), lycopene group (248.33 ± 5.11), and the α-tocopherol group (208.03 ± 4.12) when compared with the TCM group (176 ± 2.71). The diffuse distribution of mitochondria, as shown in the IGF-1 and lycopene groups in Figure 3, was significantly (p < 0.01) higher in the IGF-1 and lycopene groups (90% and 70%, respectively) when compared with the α-tocopherol group (30%). The semi-diffused mitochondrial distribution (shown in the control group in Figure 3) of the α-tocopherol group was significantly (p < 0.01) higher (40%) than that of the IGF-1, lycopene, and control groups (30%, 30%, and 10%, respectively) (Table 5, Figure 3). Conversely, the peripheral distribution of mitochondria was observed in the α-tocopherol group in Figure 3 (30%), significantly (p < 0.01) higher than in the other groups (no peripheral distribution, 0.0%) (Table 5, Figure 3).

**Table 4:** Effect of IGF-1, lycopene, and α-tocopherol supplementation on mitochondrial intensity (mean ± standard error) of *in vitro*-matured buffalo oocytes.

Groups	Number of oocytes imaged	Mean ± SE
Control	30	176 ± 2.71^d^
IGF-1	30	290.72 ± 10.19^a^
Lycopene	30	248.33 ± 5.11^b^
α-tocopherol	30	208.03 ± 4.12^c^

Replicates = 3. a,b,c,d Means within the same column bearing different superscripts differ significantly (*p* < 0.01). IGF-1 = insulin-like growth factor-1, PB = polar body, ng = nanogram, μM = micromolar.

**Table 5:** Effect of IGF-1, lycopene, and α-tocopherol supplementation on mitochondrial distribution (number and percentage) of *in vitro*-matured buffalo oocytes.

Groups	Matured oocytes	Diffused n (%)	Semi-diffused n (%)	Peripheral n (%)
Control	30	27 (90)^a^***	3 (10)^c^**	0 (0)^b^*
IGF-1	30	21 (70)^b^***	9 (30)^b^**	0 (0)^b^*
Lycopene	30	21 (70)^b^***	9 (30)^b^**	0 (0)^b^*
α-tocopherol	30	9 (30)^c^**	12 (40)^a^***	9 (30)^a^*

Replicates = 3. ***, **, * Significant difference within the same row (p < 0.01). a,b,c Means within the same column bearing different superscripts differ significantly (*p* < 0.01). IGF-1 = insulin-like growth factor-1, PB = polar body, ng = nanogram, μM = micromolar.

## DISCUSSION

### Effect of IGF-1, lycopene, and α-tocopherol supplements to the maturation medium on the rate of maturation of buffalo oocytes

#### Cytoplasmic maturation

Our study revealed that lycopene improves the cytoplasmic maturation rate of buffalo oocytes when compared to the IGF-1, α-tocopherol, and control groups. Lycopene increased cytoplasmic maturation in buffalo oocytes due to its mechanism of action, which stimulated the continued maintenance of cumulus cells and oocyte gap junctional communication [40]. Lycopene aids in the stabilization of connexin43 mRNA [41], resulting in effective cytoplasmic maturation. The result for IGF-1 is consistent with the previous studies, which reported no significant difference between the control and IGF-1 groups in buffalo [7, 42]. This finding is due to the mechanism of action of IGF-1, which does not function through cumulus cells (CC) or obstruct the oocyte’s ability to produce an expansion factor in bovine oocytes [43]. In addition, the result of α-tocopherol is that it does not affect cumulus expansion in buffalo [44] because it is not responsible for cumulus expansion in porcine [45].

#### Nuclear maturation

In this study, IGF-1 and lycopene improved the oocyte nuclear maturation rate in buffalo compared with the α-tocopherol and control groups. The IGF-1 results agree with those of Pawshe *et al*. [46], who demonstrated that 100 ng/mL IGF-1 is a major follicular factor responsible for enhancing maturation of buffalo oocytes (79.0%) compared with the control (TCM-199, 34.9%). Ismail *et al*. [7] showed a significantly better increase in the rates of nuclear maturation of buffalo oocytes that were matured *in vitro* with IGF-1 (range 81.21–86.56%) compared with the control (range 70.18–73.48%) in buffalo. In contrast, some authors did not find the beneficial effects of IGF-1 treatment during *in vitro* production of bovine embryos [47–49]. Once IGF-1 is released, it functions in a paracrine and autocrine manner on GC, influencing oocyte maturation [50]. The expression levels and localization of IGF-1, IGF-II, IGFBP-4, IGFBP-5, and the type II receptor in follicular cells vary across mammalian species, suggesting potential species-specific roles in ovarian folliculogenesis. Administering 100 ng/mL IGF-1 stimulates the activation of primordial follicles and decreases DNA fragmentation via the PI3K/AKT pathway [51]. Maturation with IGF-1 begins when its membrane receptor is activated, requiring the tyrosine dephosphorylation of p34, a component of the maturation-promoting factor (MPF) [52]. In bovine oocytes, MPF activation coincides with germinal vesicle breakdown (GVBD) and is responsible for phosphorylating proteins involved in nuclear membrane formation, chromatin condensation, and microtubule organization [53]. H1 kinase activity, which correlates with IGF-1 supplementation in bovine oocytes, increases rapidly, indicating meiotic progression through PB extrusion [54]. *In vitro* studies have shown that IGF-1 synergizes with FSH to regulate the aromatase activity of GC [55]. IGF-1 is essential for controlling cell survival, proliferation, and steroidogenesis in GC. IGF-1 and IGF-II intrafollicular concentrations control estradiol E2 production [56]. In addition, IGF-1 increased BCL2 gene expression in bovine oocytes and COX-2 gene expression in CC [57]. Additionally, the lycopene group agreed with Chowdhury *et al*. [58] and Sidi and Residiwati [12], whose results demonstrated that lycopene had a higher nuclear maturation rate than the control group when supplemented to the maturation medium of bovine oocytes. Lycopene partially maintained the developmental competence of oocytes subjected to oxidative damage induced by menadione by reducing ROS levels [13]. In mammals, mitogen-activated protein kinase (MAPK) signaling is essential for meiotic development, microtubule organization, spindle formation, and chromosomal segregation [59]. Lycopene may promote the resumption of meiosis because it has been shown to activate MAPK in various cell types. In contrast, in porcine [40], whose results revealed that lycopene supplementation into IVM did not affect meiotic competence but significantly increased the glutathione level of mature porcine oocytes. The results of α-tocopherol agreed with Thiyagarajan and Valivittan [16], who revealed that α-tocopherol had no significant effect on nuclear maturation when supplemented in the buffalo oocyte maturation medium. Azam *et al*. [44] revealed that supplementation of α-tocopherol to maturation media containing α-linolenic acid did not enhance *in vitro* oocyte maturation in buffalo, and Tao *et al*. [45] revealed that α-tocopherol has little efficacy on the nuclear maturation of matured oocytes of porcine due to the existence of the surrounding CC. Adeldust *et al*. [60] demonstrated that α-tocopherol supplementation to the maturation media did not have favorable effects on ovine oocyte maturation *in vitro*. According to Dalvit *et al*. [22], bovine cumulus–oocyte complexes (COCs) showed a 50% decrease in the naturally occurring α-tocopherol content in their membranes during *in vitro* maturation of bovine oocytes, suggesting a partial loss of antioxidant action during the *in vitro* culture phase. Consequently, the beneficial impact of additional α-tocopherol supplementation in the IVM media on the oocyte maturation rate can be attributed to the fact that α-tocopherol defends the polyunsaturated fatty acids in membranes against free radicals. Lipid-soluble α-tocopherol helps preserve cell viability by being more evenly distributed throughout the lipid-rich environment of the oocyte. α-tocopherol may also help maintain GSH synthesis in porcine oocyte CC by preventing DNA fragmentation [45].

### Effect of IGF-1, lycopene, and α-tocopherol supplements to IVM medium on developmental competency of mature buffalo oocytes

According to our findings, the IGF-1 and lycopene groups show significantly higher cleavage, morula, and blastocyst rates (p < 0.01) than the control and α-tocopherol groups. The results of IGF-1 are consistent with those of Ismail *et al*. [7], whose study revealed that IGF-1 significantly increases the rates of cleavage, morula formation, and blastocyst formation in *in vitro*-matured buffalo oocytes. And agreed with [61, 62] in bovine studies. In yak-cattle crossbred embryos, IGF-1 increased blastocyst rates (p < 0.01), but the cleavage rate was not affected [63]. Fernandez-Gonzalez *et al*. [64] showed that IGF-1 supplementation increases the morula and blastocyst rate in the cat. IGF-1 induces better nuclear maturation and supports embryonic development to the blastocyst stage in buffalo [65]. IGF-1 may regulate apoptosis by stimulating protein synthesis in bovine GC [66]. Lycopene significantly increased (p < 0.01) the rates of cleavage, morula formation, and blastocyst formation in contrast to the control group, which aligns with the results of Chowdhury *et al*. [58], who revealed that lycopene improves blastocyst quality in IVC systems by reducing intracellular ROS concentrations and apoptosis, as well as enhancing the upregulation of the anti-apoptotic gene BCL2 and downregulation of NF-kB, COX-2, iNOS, and BAX genes in bovine. Residiwati *et al*. [14] and Sidi *et al*. [13] demonstrated that lycopene supplementation during bovine oocyte maturation improves blastocyst rate and embryo cell quality (total cell, trophectoderm, and ICM numbers) by decreasing oocyte ROS production levels, but lycopene did not affect the cleavage rate. Biswas *et al*. [26] revealed that lycopene supplementation during IVC improved porcine embryonic development by controlling mitochondria-dependent apoptosis and oxidative stress. Lycopene outperforms IGF-1 in blastocyst development. Previous buffalo studies have suggested that IGF-1 was superior. This study reveals that lycopene is superior to IGF-1 for blastocyst yield. These findings reverse the earlier understanding and provide new biological insights. The results of α-tocopherol coincide with those of Thiyagarajan *and* Valivittan [16], who revealed no significant effect of α-tocopherol on the developmental competency of buffalo oocytes during IVM. According to Azam *et al*. [44], adding α-tocopherol to maturation media containing α-linolenic acid and in embryo culture media did not improve the *in vitro* embryonic development of buffalo. Dalvit *et al*. [22] reported that α-tocopherol did not significantly affect developmental competence compared with the control in bovine and sheep [60]. In contrast, Thiyagarajan and Valivittan [16] revealed that culture under 20% O_2_ enhanced *in vitro* embryo developmental competency in buffalo by shielding them from oxidative stress and increased the frequency of blastocyst formation by 18.79% compared with the control (9.23%) and total cell count by 116.50% compared with the control (87.16%). Furthermore, it can improve embryonic quality in sheep [20], whose results indicated that supplementing with 200 μM α-tocopherol at a 20% O_2_ level improves the *in vitro* embryonic developmental competence in ovine by shielding them from oxidative stress. Báez *et al*. [17] showed that dilution of α-tocopherol in ethanol (0.05%) during IVM in bovine reduced apoptosis and improved SOD2 expression. These differences may be due to differences in the breed and the α-tocopherol doses. α-tocopherol shows an unusual distribution pattern in the peripheral mitochondria. This is also new and has not been previously reported in buffalo. This study demonstrates that alpha-tocopherol improves mitochondrial intensity but induces peripheral clustering, a potential developmental limitation. This helps explain why alpha-tocopherol does not enhance blastocyst formation.

### Effect of IGF-1, lycopene, and α-tocopherol supplementation to IVM medium on mitochondrial function in buffalo oocytes

This work is the first to quantify mitochondrial distribution in buffalo oocytes treated with lycopene and IGF-1, addressing a significant gap in buffalo-specific mitochondrial research. There is limited information available regarding the potential benefits of antioxidant supplementation to the IVM medium on mitochondrial intensity and distribution in buffalo oocytes. The novel contribution of this study is the analysis of the mitochondrial profile through a quantitative comparison of mitochondrial intensity. Distribution categories include diffuse, semi-diffuse, and peripheral, assessed using confocal imaging for each treatment. This study found that mitochondrial intensity in the IGF-1, lycopene, and α-tocopherol groups was significantly higher (p < 0.01) than in the control group and showed the highest mitochondrial diffusion distribution, compared with the semi-diffuse and peripheral distributions across all groups. Mitochondrial distribution in equine oocytes changes dynamically during maturation. The distribution is relatively uniform in the early stages, but shifts to a heterogeneous, clustered pattern by the final MII stage. This heterogeneous distribution, characterized by a concentration of mitochondria in the inner cytoplasmic region and minimal presence in the cortex, is considered an indicator of oocyte quality and maturity. This arrangement supports the high energy demands required for fertilization and early embryonic development [67]. IGF-1 improves mitochondrial function in the current study, which agrees with the findings of Ascari *et al*. [68] and Ispada *et al*. [69]. In bovines, IGF-1 increased mitochondrial membrane capacity by regulating the expression of the cytochrome C oxidase subunit 1 (COX-1) gene, thereby indirectly affecting oocyte metabolism and respiratory chain activity. They also reduce ROS generation and inhibit cytochrome C release [70], thereby decreasing endothelial cell apoptosis [71]. IGF-1 enhances mitochondrial polarization and the main mitochondrial markers and promotes ATP production through a PI3K/Akt-signaling pathway. The improvement in steroid biosynthesis and progress through the G2-M1 phase transition support the importance of energized and active mitochondria in zebrafish [25]. Lycopene also increases mitochondrial activity, as confirmed by Sidi *et al*. [13], who demonstrated that lycopene enhances mitochondrial activity by reducing ROS levels in bovine oocytes. Lycopene reduced cytochrome c release, enhanced the potential of mitochondrial membranes, prevented caspase 3 activation, and inhibited intracellular ROS production, all of which decreased apoptosis in porcine oocytes [26]. Moreover, the same authors revealed that lycopene improved mitochondrial function and protection. Mitochondrial superoxide dismutase (SOD2), a member of the iron/manganese superoxide dismutase family, is known as manganese-dependent superoxide dismutase (MnSOD) that participates in oxidative stress and apoptotic signaling and neutralizes ROS generated during oxidative stress [72]. In contrast, lycopene supplementation did not affect the IVM of bovine oocytes in terms of oocyte mitochondrial distribution and activity or cortical granule migration and distribution [73]. These differences may be attributed to variations in lycopene doses or breeds. Maintaining the oxidative stability of membrane-bound lipids and preventing damage from reactive oxygen species depend heavily on tocopherol’s location within the mitochondrial membranes, which are the primary sites of oxidative processes and ROS generation [74]. In this study, alpha-tocopherol was found to enhance mitochondrial activity in *in vitro*-matured buffalo oocytes, which aligns with findings emphasizing its antioxidant effects. It scavenges free radicals more quickly than fatty acid side chains or membrane proteins. This process disrupts the lipid peroxidation chain and increases embryonic developmental competency in buffalo [16]. Maintaining the oxidative stability of membrane-bound lipids and preventing damage by ROS require the incorporation of α-tocopherol into mitochondria and other cellular compartments [27]. The differences in *in vitro* embryo development and mitochondrial function among various animals may arise from breeding differences, seasonal variation, semen batches, the doses of the compound used, the culture media, or the chemical composition and batches of the supplements. Moreover, mitochondrial analysis methods in oocytes and embryos are limited. To the best of our knowledge, this is the first report linking oocyte mitochondrial dynamics to embryonic developmental competence in buffalo. This study shows a clear correlation: higher mitochondrial intensity → higher cleavage/morula/blastocyst rates. This relationship has not been previously demonstrated in the study of buffalo oocytes.

## CONCLUSION

The present study demonstrated that supplementation of IVM medium with lycopene (0.2 μM) significantly enhanced cytoplasmic maturation through superior cumulus cell expansion (Grade III: 58%, GII: 28.7%; p < 0.01), nuclear maturation (87.3%; p < 0.01), and blastocyst formation compared with control, IGF-1, and α-tocopherol groups. IGF-1 (100 ng/mL) also significantly improved nuclear maturation (85.2%; p < 0.01), cleavage, morula, and blastocyst rates (p < 0.01). All three supplements increased mitochondrial intensity (p < 0.01), with IGF-1 and lycopene promoting a desirable, diffuse mitochondrial distribution, whereas α-tocopherol induced peripheral clustering and showed limited developmental benefits. Lycopene outperformed IGF-1, particularly in blastocyst yield, contradicting previous buffalo-specific findings.

These findings provide a practical, cost-effective strategy for improving oocyte quality and blastocyst production rates in buffalo *in vitro* embryo production systems. Lycopene and IGF-1 supplementation can be readily adopted in commercial and research laboratories to enhance the efficiency of assisted reproductive technologies, accelerate genetic improvement programs, and support germplasm conservation in Bubalus bubalis, particularly in regions with large buffalo populations such as Egypt.

The major strength of this work is its comprehensive, multi-parameter evaluation (cytoplasmic and nuclear maturation, fertilization, embryo development to the blastocyst stage, and quantitative confocal assessment of mitochondrial intensity and distribution) conducted across three independent experiments with adequate replication. To the best of our knowledge, this is the first study to quantify mitochondrial distribution patterns in buffalo oocytes treated with lycopene, IGF-1, and α-tocopherol and to directly link these mitochondrial profiles to developmental competence.

The study was conducted exclusively *in vitro* using ovaries collected postmortem from abattoir-slaughtered animals and semen from a single proven-fertile bull. Only single concentrations of each supplement were tested, and potential synergistic effects of combined supplementation were not evaluated. Molecular mechanisms (e.g., gene expression changes in BCL2, COX-2, and SOD2) were not fully investigated, and *in vivo* developmental competence of the produced embryos was not assessed.

Future studies should investigate optimal dose combinations of lycopene and IGF-1, explore transcriptomic and epigenetic profiles in treated oocytes and embryos, and validate these findings through in vivo embryo transfer trials and assessments of pregnancy rates. Comparative experiments across different buffalo breeds, seasons, and oxidative stress conditions would further strengthen applicability and elucidate the underlying signaling pathways (PI3K/AKT, MAPK).

In conclusion, lycopene emerges as the most promising supplement for enhancing IVM, mitochondrial function, and embryo developmental competence in buffalo oocytes, primarily through superior antioxidant protection and maintenance of optimal mitochondrial distribution. This study provides new biological insights, reverses prior assumptions regarding IGF-1 superiority in buffalo, and offers practical strategies to overcome the inherent low efficiency of *in vitro* embryo production in this economically important species.

## DATA AVAILABILITY

The datasets generated during the current study are available from the corresponding author upon reasonable request.

## AUTHORS’ CONTRIBUTIONS

OK: Conceptualized the study, designed the methodology, supervised the project, provided facilities, performed confocal microscope imaging, drafted the manuscript, and revised the manuscript. SE: Collected samples, conducted laboratory experiments, curated the data, analyzed the data, drafted the manuscript, and revised the manuscript. ME, SH, and NB: Analyzed the data, supervised aspects of the study, drafted the manuscript, and revised the manuscript. All authors read and approved the final manuscript.
